# Combining statistical methods for detecting potential outliers in groundwater quality time series

**DOI:** 10.1007/s10661-022-10661-0

**Published:** 2022-11-08

**Authors:** Wilbert Berendrecht, Mariëlle van Vliet, Jasper Griffioen

**Affiliations:** 1 Berendrecht Consultancy, Stakenbergerhout 107, Harderwijk, 3845 JE the Netherlands; 2 TNO Geological Survey of the Netherlands, P.O. Box 80015, Utrecht, 3508 TA the Netherlands; 3 Copernicus Institute of Sustainable Development, Faculty of Geosciences, Utrecht University, Utrecht, the Netherlands

**Keywords:** Outlier detection, Non-detects, Detrending, Monitoring networks, Groundwater quality data

## Abstract

Quality control of large-scale monitoring networks requires the use of automatic procedures to detect potential outliers in an unambiguous and reproducible manner. This paper describes a methodology that combines existing statistical methods to accommodate for the specific characteristics of measurement data obtained from groundwater quality monitoring networks: the measurement series show a large variety of dynamics and often comprise few (< 25) measurements, the measurement data are not normally distributed, measurement series may contain several outliers, there may be trends in the series, and/or some measurements may be below detection limits. Furthermore, the detection limits may vary in time. The methodology for outlier detection described in this paper uses robust regression on order statistics (ROS) to deal with measured values below the detection limit. In addition, a biweight location estimator is applied to filter out any temporal trends from the series. The subsequent outlier detection is done in z-score space. Tuning parameters are used to attune the robustness and accuracy to the given dataset and the user requirements. The method has been applied to data from the Dutch national groundwater quality monitoring network, which consists of approximately 350 monitoring wells. It proved to work well in general, detecting outliers at the top and bottom of the regular measurement range and around the detection limit. Given the diversity exhibited by measurement series, it is to be expected that the method does not give 100% satisfactory results. Measured values identified by the method as potential outliers will therefore always need to be further assessed on the basis of expert knowledge, consistency with other measurement data and/or additional research.

## Introduction

Monitoring is an indispensable part of the management of the availability or quality of water resources, whether surface water or groundwater. Monitoring comprises a series of steps that starts with definition of the monitoring objectives and finishes with the evaluation of the results and potential optimisation of the network if monitoring carries on as a continuous process (Rentier et al., 2006; Van Geer et al., 2008; Ward et al., 2004). An important step is the quality assurance and quality control. Quality assurance comprises the a priori prevention of errors in installation, sampling, measurement, etc.; quality control entails the a posteriori detection of such errors (Ferretti, 2009). Both are needed, as errors can never be excluded.

In water resources management, monitoring networks are commonly established by bodies such as water authorities or drinking water companies. A distinction is made between state monitoring and trend monitoring. In state monitoring, comparisons are only possible spatially, for a certain moment in time. Trend monitoring, however, enables intercomparison of individual measurements with preceding and subsequent measurements from the same observation point (Broers & Van der Grift, 2004). It is used, for example, to distinguish seasonal trends from multi-annual trends in groundwater level and to investigate the efficiency of measures for reducing water pollution. The monitoring frequency is determined by the temporal dynamics and cost. The highest frequencies are usually applied when monitoring river levels, as these have high temporal dynamics; the lowest frequencies are used to monitor the quality of deep groundwater, which has the lowest temporal dynamics. In general, groundwater quality monitoring networks have a relatively low monitoring frequency, as sampling and analysis are expensive and the dynamics are usually not large. This implies that the data density will also be low.

Quality control of monitoring networks requires detection of artificial outliers, as they may indicate bad data and influence interpretation of the monitoring data. The low temporal density of groundwater quality monitoring networks makes this a challenging task: these networks may be only a few decades old (exceptions are long-established monitoring networks at sites where groundwater is abstracted to provide drinking water) and their monitoring frequency may be annually or even less frequently for financial reasons. Additionally, the measurements in groundwater quality monitoring are frequently below detection limit because of the limitations of the measurement process or analytical technique. These so-called non-detects are too uncertain to be considered reliable.

An outlier could be generally defined as being a measurement (or subset of measurements) which appears to be inconsistent with the remainder of the dataset (Barnett & Lewis, 1994). Here, inconsistency can mean that the measurement is from a different distribution than the model or distribution considered to describe the data. But inconsistency could also mean that the presupposed model or distribution is not describing the data as well as was assumed (Zimek & Filzmoser, 2018). In terms of water quality data, the latter means that an analytically correct measurement could be identified as an outlier. For this reason, in this paper we apply outlier detection methods to identify potential outliers, i.e., measurements that are on some objective statistical criterion inconsistent with the rest of the sample. This enables us to perform quality control on large datasets in an automated procedure. Whether a potential outlier is a true outlier or not should then be decided based on additional information or checks.

Tests that have been proposed for detecting outliers consider a criterion based on (1) the interquartile range (Tukey, 1977), (2) the standard deviation or (3) a range defined by the median plus or minus a multiplication of the median absolute deviation (Hodge & Austin, 2004; Walfish, 2006). The first two types of tests assume normality, but groundwater quality data are often not normally distributed. The second type does not work properly for small datasets (Cousineau & Chartier, 2010; Leys et al., 2013). It does not assume normality and is commonly called the MAD or Hampel identifier test (Hampel, 1972). All these tests have difficulty handling series of analyses that lie close to 0, i.e. that vary around the analytical detection limit, which is typical for major redox-sensitive ions such as iron and nitrate, and for trace elements and micro-organics. Tests designed specifically for small datasets, such as Dixon’s Q test (Dean & Dixon, 1951) and Grubbs’ test (Grubbs & Beck, 1972), are sensitive for masking, which means that if several outliers are present, one may escape detection (Acuna & Rodriguez, 2004; Barnett & Lewis, 1994; Bendre & Kale, 1987). They are therefore especially suitable when only one outlier is present, but they cannot handle data with values below detection limit, which is a typical aspect of water analysis. Another major shortcoming is that Grubbs’ test assumes normality.

Reijnders et al. (2004) describe a method to identify potential outliers in multi-annual time series (< 25 years) of the Dutch National Groundwater Quality Monitoring Network. Extreme values were detected with a moving-average approach where every value was compared to the two surrounding values. This seems to be a fruitful approach for dealing with temporal trends. However, such a moving-average estimator is not robust in a sense that it is sensitive to extreme values (Hippke et al., 2019). In addition, the method of Reijnders et al. (2004) cannot handle values below detection limit.

The objective of our study was to present a method to detect potential outliers by combining the moving-window approach inspired by Reijnders et al. (2004) with a method to handle values below varying detection limits (non-detects), and an outlier labelling method to flag potential outliers (Kannan et al., 2015). The moving-windows approach is based on Tukey’s biweight filter (Mosteller & Tukey, 1977; Stock & Watson, 2012), which was found to be an effective and robust filter for detrending by Hippke et al. (2019). Non-detects are handled by applying a robust regression on order statistics (ROS) estimator as presented by Helsel (2005). The ROS estimator can deal with multiple detection limits and performs well for small data sets as well as large ones (Baccarelli et al., 2005; Helsel, 2006). We demonstrate the methodology established using a dataset obtained from the Dutch national groundwater quality monitoring network, which was set up in the early 1980s and consists of circa 350 wells spread across the Netherlands. Results show that the method identifies potential outliers well in the presences of trends and/or various detection limits.

## Methods and materials

### Starting points

Groundwater quality measurement series tend to have the following six properties: (1) they contain relatively few measurement points (< 25); (2) trends may be present in the series; (3) the data are not normally distributed; (4) each series may contain an unknown number of outliers; (5) measured values may be below a detection limit (non-detects); and (6) detection limits vary over time. We have therefore developed a method for detecting an unknown number of outliers in data with possible trends and non-detects that take these six properties into account as well as possible. The method is based on (1) estimating values below detection limit; (2) log-transformation of the dataset to improve the symmetry of the distribution; (3) removing trends in the measurement series by using a low-pass filter; (4) calculating the deviations from the median for each measurement series; (5) merging data from multiple measurement series to produce more robust statistics; (6) including information on detection limits when determining whether a measurement qualifies as a potential outlier; and (7) retrospectively assessing potential outliers using visual inspection and additional information.

### Dealing with values below detection limit

Aqueous solutes may be present below the detection or reporting limits, resulting in values reported as a non-detect or less-than. As outlier detection methods are generally based on sample statistics, a method is required to replace a non-detect with a representative substitution enabling the calculation of statistics such as the mean, median and standard deviation. A widely applied method for estimating sample statistics in the presence of non-detects is the robust regression on order statistics (ROS) estimator (Helsel, 2005; Shumway et al., 2002). ROS is a semi-parametric method in which non-detects are replaced on the basis of least-squares regression on a probability graph. The method divides non-detects with a common detection limit such that each is an equal part of the probability distribution under the detection limit. For a detailed description of the methodology, see e.g. Helsel (2005) and Lee and Helsel (2005).

The ROS estimator is commonly recommended in the literature over the widely used direct substitution method (Helsel, 2006; Helsel & Cohn, 1988; Singh & Nocerino, 2002). The latter method replaces all non-detects with, say, 0, the detection limit (DL), or half the detection limit DL/2 (Helsel, 1990). However, Sinha et al. (2006) recommends the ROS method only if less than 50% of the measurements are below the detection limit. The following two criteria were therefore chosen to determine whether to apply the ROS estimator for a measurement series:

1. The measurement series must contain at least five detected values
2. At least 50% of the measured values must have been detected

If these two criteria are not met, we continue to use direct substitution (DL/2). After any non-detects have been replaced with an estimated value, the sequences are log-transformed.

### Detrending

Measurement series of groundwater quality data may exhibit trends. This complicates the detection of outliers, because trends affect the statistical distribution around the mean or median. It is therefore better to remove the trend from the measurement series prior to outlier detection. One method of doing this is by estimating a local mean for the measurement series. The outlier detection is then performed on the deviation from this local mean. Stock and Watson (2012) apply the biweight location as an estimator for the local average. The biweight location can be considered a low-pass filter and is a robust statistic for determining the central location of a distribution. It is described as (Beers et al., 1990):

1 $${\upzeta }_{\mathrm{biloc}}=\mathrm{M}+\frac{{\sum }_{\left|{\mathrm{u}}_{\mathrm{i}}\right|<1}\left[\left({x}_{i}-M\right){\left(1-{u}_{i}^{2}\right)}^{2}\right] }{{\sum }_{\left|{\mathrm{u}}_{\mathrm{i}}\right|<1}{\left(1-{u}_{i}^{2}\right)}^{2}}$$

with ${x}_{i}$ the measurement data, $M$ the sample median, and ${u}_{i}$ calculated as:

2 $${u}_{i}=\frac{\left({x}_{i}-M\right)}{c*{\text{MAD}}}$$

with $c$ the *tuning* constant and ${\text{MAD}}$ the median absolute deviation. If ${\text{MAD}}$ is zero, the median is used as estimator for the central location. Typical values for $c$ are 6.0 or 9.0. A lower $c$ value yields a more robust estimate (less sensitive to outliers), a higher $c$ value yields a more efficient estimate (better approximation of the maximum likelihood estimator). The essence of the biweight location estimator is that points further from the sample median are given less weight, and values for a ${\text{MAD}}>c$ are not included.

The biweight location is calculated across a central window of seven measured values, i.e. three measurements on either side of the measurement itself are used. The calculation must be adjusted for measurements at the beginning and end of the measurement series. For ${t}_{i}<{t}_{3}$ a window of ${t}_{0},\dots , {t}_{i+2}$ is used, and the window for ${t}_{i}>{t}_{N-2}$ is ${t}_{i-2},\dots , {t}_{N}$, where N is the number of measurements in the series. Since the monitoring networks for groundwater quality are generally sampled at regular intervals (e.g. annually), we opted not to take the actual time interval between the measurement points into account.

**Fig. 1:**
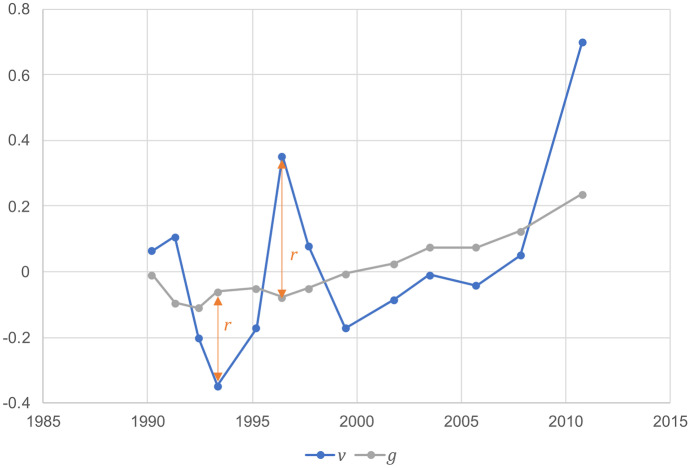
Calculation of deviation $r=v-g$ relative to biweight location

The deviation between the measured value ${v}_{i}$ at time ${t}_{i}$ and the local mean ${g}_{i}$ at time ${t}_{i}$ is now calculated as (see Fig. 1):

3 $${r}_{i}={v}_{i}-{g}_{i}$$

If the measured value ${v}_{i}$ lies below the detection limit, the imputed value is used.

### Calculating modified z-scores per measurement series

The modified z-score is a standardised score that measures outlier strength, i.e., how much a certain score differs from the typical score (Iglewicz & Hoaglin, 1993). The modified z-score is more robust than the standard z-score because it uses the median rather than the mean. The modified z-score for measured values $i$, ${M}_{i}$, is calculated here as the deviation of ${r}_{i}$ from the median of $r$, weighted by the series mean absolute deviation from the median and a scaling factor:

4 $${M}_{i}=\frac{{r}_{i}^{\mathrm{^{\prime}}}}{1.2533*\mathrm{MAD}}$$

where ${r}_{i}^{^{\prime}}=\left({r}_{i}-\widetilde{r}\right)$ and $\widetilde{r}$ is the median of $r$, and:

5 $$\mathrm{MAD}=\frac{1}{N}{\sum }_{i}^{N}\left|{r}_{i}^{\mathrm{^{\prime}}}\right|$$

The scaling factor 1.2533 is such that for a large number of normally distributed measurements (whether or not they are log-transformed), the value MAD/scaling factor approaches the standard deviation of the measurements. In this paper, we have opted to work with the mean deviation rather than the median deviation from the series median. In series with a small number of measurements and/or series with little variation or many values below the detection limit, the median deviation tends to be too low, and hence the z-score of measured values soon becomes high, which results in relatively many measured values being marked as potential outliers. A disadvantage of basing the z-score on the mean deviation is that if a series has multiple outliers, it is more difficult to detect all of them.

For each measurement series $k$ with at least seven detected values, the following typical series score ${s}_{k}$ is calculated:

6 $${s}_{k}=\frac{{\mathrm{MAD}}_{k}}{1.2533}$$

If the values below the detection limit have been imputed using the ROS method, then all detects and non-detects are used to calculate the above score. If non-detects have been imputed as DL/2, then only the detected measurement values are included.

### Detecting outliers by using the composite z-score

When the measurement series consists of a small number of measurements, the z-score is sensitive to “coincidences”. To increase the robustness of the z-score, the complete set of measurement series from a monitoring network is merged, per parameter, based on the calculated score ${s}_{k}$. For this purpose, the data of all $N$ measurement series are sorted by ${s}_{k}$ ($k=1,\dots ,N)$ and divided into 10-percentile ranges (Reijnders et al, 2004), i.e. P_0_-P_10_, P_10_-P_20_, P_20_-P_30_, etc. Next, the mean and standard deviation are calculated over all detrended values ${r}_{k,i}^{\mathrm{^{\prime}}}$ within a 10-percentile range. This yields a value for $s,$ plus the corresponding standard deviation for $r\mathrm{^{\prime}}$, ${\sigma }_{r\mathrm{^{\prime}}}$ for each 10-percentile range. These values are then plotted against each other in a graph. On the basis of the points, linear regression is then used to estimate a linear relationship between ${s}_{k}$ and the standard deviation $\widehat{\sigma }$:

7 $$\widehat{\sigma }=a*{s}_{k}+b$$

where $a$ and $b$ are linear regression parameters. Finally, a threshold value for $\widehat{\sigma }$, ${\sigma }_{\text{min}}$, is applied:

8 $${\widehat{\sigma }}^{*}={\text{max}}\left(\widehat{\sigma }, {\sigma }_{\text{min}}\right)$$

In this way, if the series has little temporal variation, a slightly different measurement will not already be considered a potential outlier. It may not necessarily be an outlier, as it might also result from variation in recharge conditions, hydrodynamic dispersion along the flow path, noise in the lab analysis, etc. The height of the threshold value ${\sigma }_{\text{min}}$ depends on the dataset and the extent to which small deviations within measurement series with limited fluctuations should be considered as potential outliers.

The “composite z-score” ${M}_{i}^{*}$ now becomes:

9 $${M}_{i}^{*}=\frac{{r}_{i}^{\mathrm{^{\prime}}}}{{\widehat{\sigma }}^{*}}$$

A measured value is considered a potential outlier if the absolute value of ${M}_{i}^{*}$ exceeds a predetermined threshold value ${M}_{crit}$. A commonly used threshold for ${M}_{crit}$ is 3.5 (Iglewicz & Hoaglin, 1993).

### Correcting for measurement values below the detection limit

The threshold value applied above concerns the variation in the measurement series (whether or not the series has been log-transformed) that is the result of natural, sampling and analytical variability. However, for very low measurement values it is also desirable to apply a threshold in relation to one or more detection limits. For some parameters, the detection limit varies over time, with the result that a value detected at one measurement moment may be lower than the detection limit at another measurement moment.

To prevent the method from detecting such low readings as outliers, a threshold value ${v}_{DL}$ is derived by making histograms of the detection threshold values per parameter. Based on these histograms, threshold values are chosen (Table 1). In most cases, the threshold value is the median value of all detection limits for a parameter. A value measured at time ${t}_{i}$ is not considered an outlier if both the measured value and the local mean at time ${t}_{i}$ are below this threshold value.

**Table 1:** Threshold values ${\mathrm{v}}_{\mathrm{DL}}$ per parameter, based on common detection limits

Parameter	Threshold value		Parameter	Threshold value	
Al	13.49	µg/l	K	1.21	mg/l
As	0.15	µg/l	Mg	0.882	mg/l
Ba	2.75	µg/l	Mn	0.002	mg/l
Ca	2	mg/l	NH_4_	0.014	mg N/l
Cd	0.045	µg/l	NO_3_	0.1	mg N/l
Cl	0.11	mg/l	Na	0.184	mg/l
Cr	0.7	µg/l	Ni	0.88	µg/l
Cu	0.7	µg/l	P-total	0.062	mg P/l
DOC	0.6	mg/l	Pb	0.207	mg/l
Fe	0.011	mg/l	SO_4_	0.1	mg/l
HCO_3_-field	0.25	mg/l	Sr	0.5	µg/l
HCO_3_-lab	3	mg/l	Zn	6.54	µg/l

### Data for validating the method

The method has been tested using data obtained from the Dutch national groundwater quality monitoring network, which was set up in the early 1980s and consists of c. 350 wells spread across the Netherlands. The wells contain three screens, with the first at c. 10 m depth, the second at c. 15 depth and the third screen at c. 25 depth (Broers, 2002; Van Duijvenbooden, 1993). Deviations in screen depth hold when clay layers were encountered at these depths during drilling.

Only the shallowest and deepest screens have been sampled. The middle screen is a back-up screen. Since 1997, a distinction in the strategy for sampling the screens has been made between one-, two- and 4-year measurement cycles (Van Vliet et al., 2012; Wever & Bronswijk, 1997). The pH, EGV, temperature, oxygen and bicarbonate (HCO_3_^-^) are determined in the field during sampling (Van Vliet et al., 2010). Macro components (NO_3_, SO_4_, NH_4_, Cl, K, Na, Mg, Ca, Fe, Mn, Total-P, DOC, HCO_3_) and inorganic micro components (Ba, Sr, Zn, Al, Cd, Ni, Cr, Cu, As and Pb) were analysed in the laboratory. The test data used to validate the method were groundwater quality data measured from the start of the monitoring network in 1984 up to the end of 2010.

## Results and discussion

### The parameter values used

The method has several parameters that need to be configured. This allows for tuning the desired behaviour of the method to properties of the dataset, making the method flexible for practical application. The following parameters can be configured:

- The tuning constant $c$ in Eq. (2). This parameter determines the smoothness of the local mean and the impact of local extremes on the estimated trend. Various tests revealed that a value of $c$ = 9.0 for the test dataset generally produced the best results;
- The threshold value for the standard deviation ${\sigma }_{\text{min}}$ in Eq. (8). This prevents small deviations from being considered as potential outliers in series with little variation. For the dataset in this article, it was empirically determined that the standard deviation associated with the 50th percentile of $s$, ${{\sigma }_{\text{min}}=s}_{50}$ gave satisfactory results;
- The threshold value of the z-score, ${M}_{crit}$, as a measure for considering an extreme value to be a potential outlier. A value of 3.5 was chosen, as recommended by Iglewicz and Hoaglin (1993).

The results presented below are based on these configurations.

### General results

The testing procedure was applied to the analytical data on principal solutes and trace metals from all observational filters of the national monitoring network. Table 2 gives an overview of certain basic data for each parameter and also shows how many measurement values were below the detection limit and how many potential outliers were detected. It can be seen that for a large number of trace metals including Al, many values are below the detection limit. For example, about 2/3 of all values measured for Cd and Cu are below the detection limit. For redox-sensitive Fe, NO_3_ and NH_4_ and total-P there are also relatively high numbers of measurements with values below the detection limit. As a result, several measurement series contain too few (less than 7) measured values for an outlier analysis to be performed.

**Table 2:** Data characteristics per parameter and number of potential outliers when *M*_*crit*_ is 3.5

**Parameter**	**No. of wells**	**No. of filters**	**No. of measurements**	**No. below detection limit**	**No. of outliers**
Al	387	796	9894	3832 (38.73%)	13 (0.13%)
As	387	795	9279	2799 (30.16%)	46 (0.50%)
Ba	387	796	9851	40 (0.41%)	83 (0.84%)
Ca	397	838	14,231	64 (0.45%)	62 (0.44%)
Cd	387	793	9141	6341 (69.37%)	10 (0.11%)
Cl	397	838	14,234	5 (0.04%)	97 (0.68%)
Cr	387	788	7838	3004 (38.33%)	11 (0.14%)
Cu	387	793	9144	5920 (64.74%)	7 (0.08%)
DOC	396	809	8081	105 (1.3%)	54 (0.67%)
Fe	391	804	10,615	1272 (11.98%)	97 (0.91%)
HCO_3_-lab	397	827	9120	521 (5.71%)	54 (0.59%)
HCO_3_-field	363	732	5151	11 (0.21%)	22 (0.43%)
K	397	838	14,224	84 (0.59%)	45 (0.32%)
Mg	397	838	14,235	11 (0.08%)	85 (0.60%)
Mn	387	796	9854	364 (3.69%)	88 (0.89%)
NH_4_	397	835	13,530	2092 (15.46%)	130 (0.96%)
NO_3_	397	838	14,236	7773 (54.6%)	17 (0.12%)
Na	397	838	14,231	1 (0.01%)	84 (0.59%)
Ni	387	792	8561	3492 (40.79%)	15 (0.18%)
P-tot	397	838	13,513	3551 (26.28%)	86 (0.64%)
Pb	387	785	5167	2749 (53.2%)	3 (0.06%)
SO_4_	397	838	14,237	1598 (11.22%)	58 (0.41%)
Sr	387	796	9839	4 (0.04%)	60 (0.61%)
Zn	387	796	9851	5343 (54.24%)	8 (0.08%)

Table 2 shows that the number of outliers per parameter is always less than 1% of the total number of measurements for that parameter. The parameters NH_4_, Fe, Mn and Ba have relatively the most outliers: over 0.8% in each case. NO_3_, Al and the trace metals Cd, Cr, Cu, Ni, Pb and Zn have fewer outliers than the other parameters: below 0.2%. This may be related to the mostly low concentrations of these parameters and/or the large number of measured values that are below detection limit.

### Examples illustrating the method’s effectiveness

**Fig. 2:**
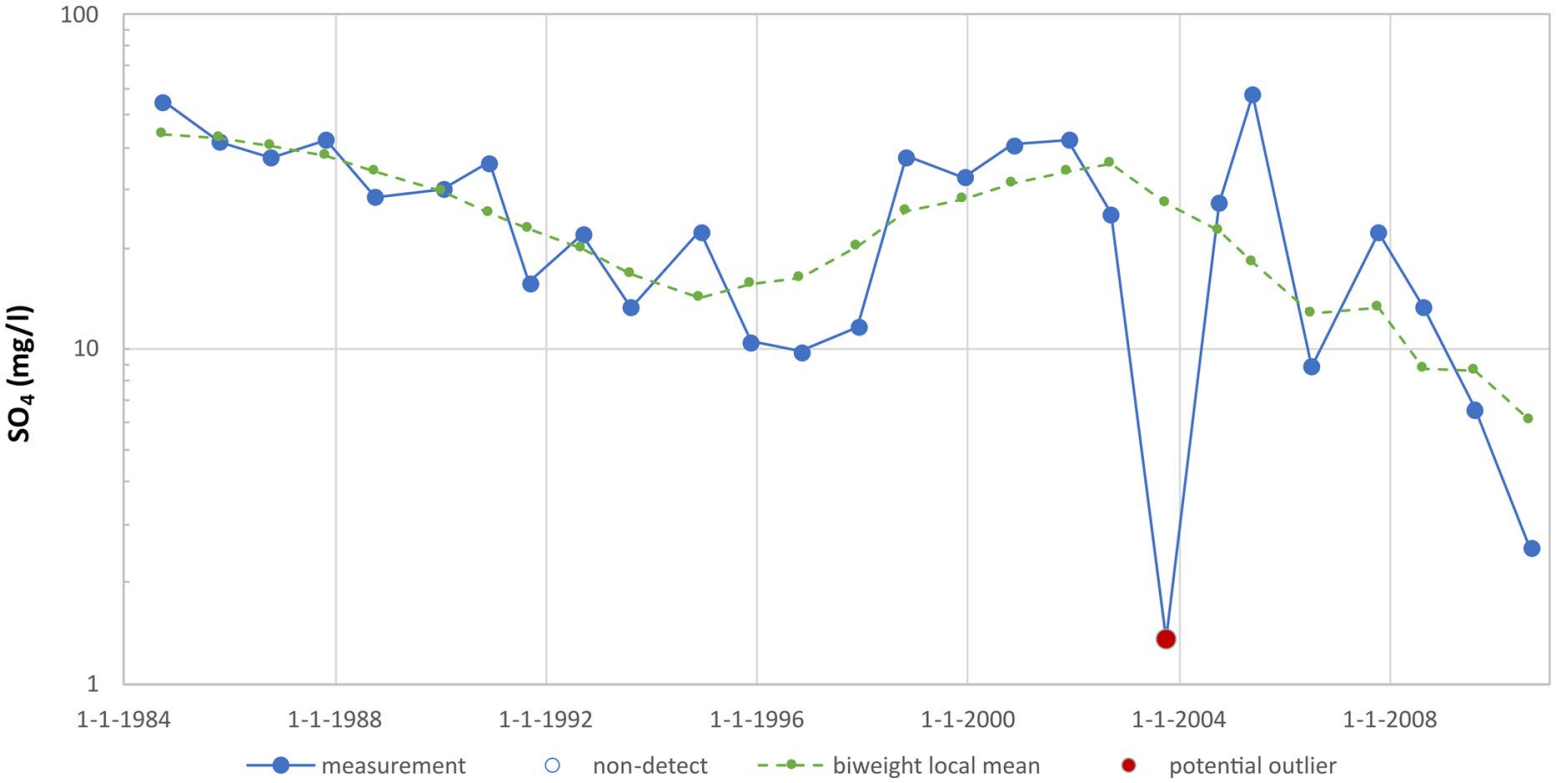
Detection of outlier in a sulphate measurement series (well 85) with downward trend, after application of biweight low-pass filter

The operation and effectiveness of the detection method are illustrated below with some examples. Figure 2 shows a measurement series for sulphate analyses. The series shows a jagged trend. The local mean follows the trend well, without being overly influenced by short-term variations. Also, due to the use of the biweight low-pass filter, the local mean is not affected by the highly anomalous value measured in 2003. Based on the deviations from the local mean, that measurement is detected as a potential outlier.

**Fig. 3:**
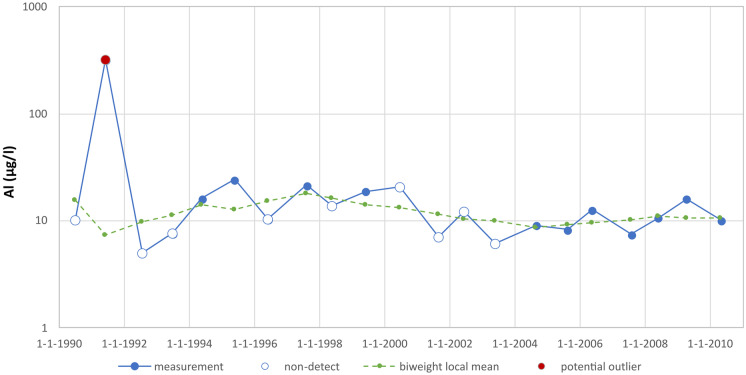
Detection of an outlier in a series of aluminium analyses (well 20) with many measured values below detection limit, which have been imputed using the ROS method

Figure 3 shows a measurement series of aluminium analyses with a detected potential outlier in 1991. Several values in the measurement series are below the detection limit. The detection limits varied over time and had values of 18.83, 13.49, 53.96 and 10 µg/l. The measured values below the detection limit were imputed using the ROS method and are shown in the figure as hollow circles. The detected and imputed values were used to derive a local mean. Using the deviations from this local mean, the z-score was calculated. The resulting z-score for the 1991 measurement was 5.5, so it has been flagged as a potential outlier.

**Fig. 4:**
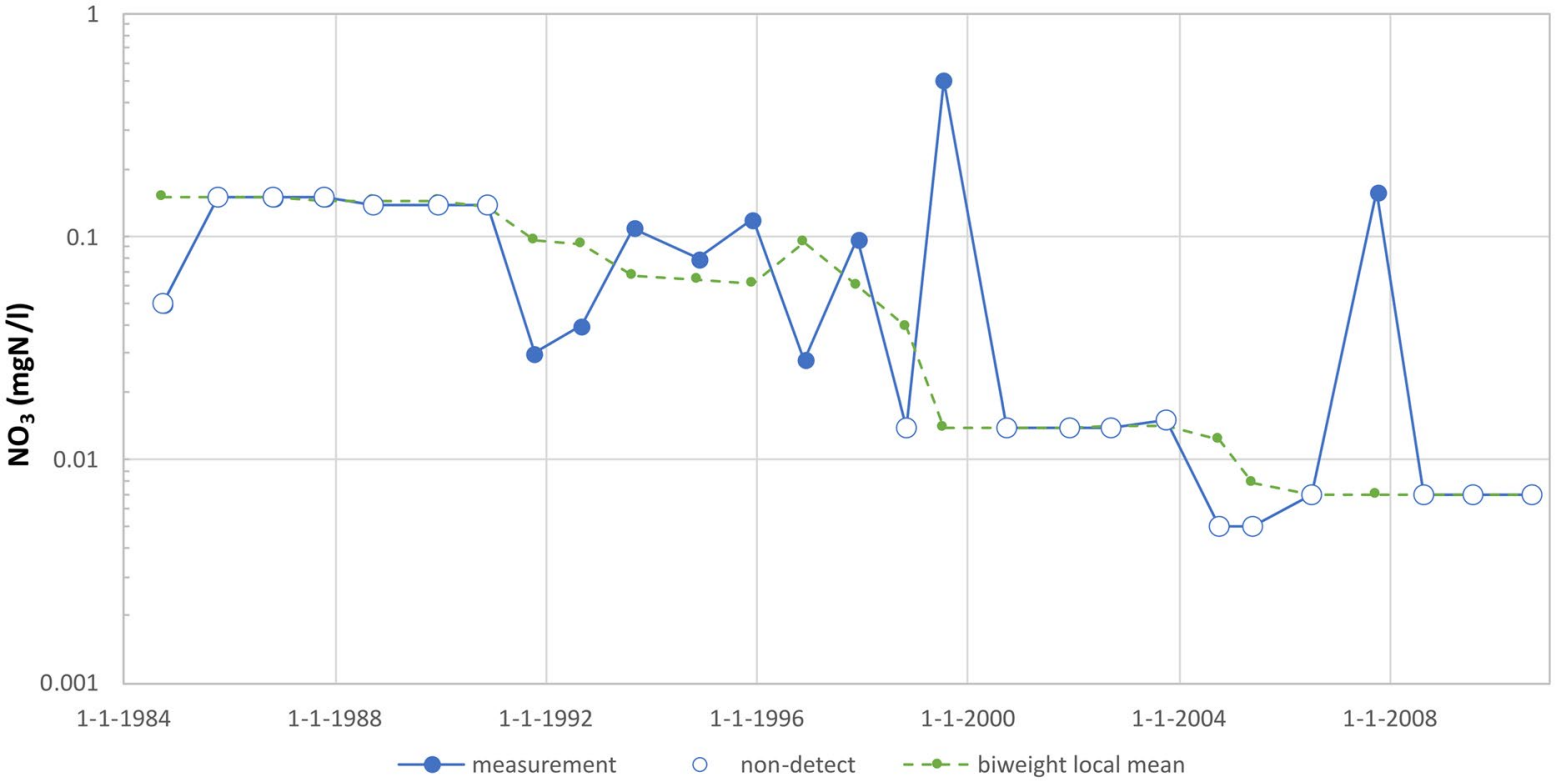
A measurement series of nitrate (well 48) in which over 50% of the values are below the detection limit. The z-score has been calculated using the detected measurements

In Fig. 4, a measurement series of nitrate is shown in which over 50% of the measured values are below the detection limit, even though the detection limit decreased substantially over time. In this case, the non-detects have been replaced by directly substituting half of the detection limit value. Consequently, as indicated in the “Detrending” section, the variation of the measurement series — and hence also the z-score — has been determined using only the detected measured values. If the non-detects had been used, the variation of the measurement series would have been greatly underestimated, thereby greatly increasing the likelihood that a measured value would be wrongly identified as a potential outlier.

**Fig. 5:**
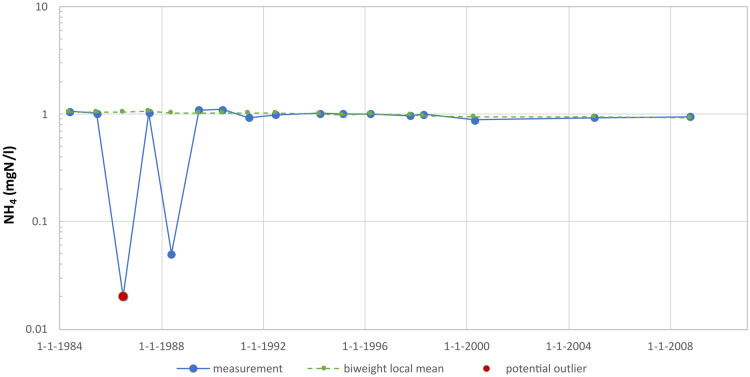
Example of an ammonium series (well 99) where the use of the mean absolute deviation (MAD) is less successful: the second low measurement in 1988 is not detected as an outlier, although from this graph it would be expected to be one

Choosing to calculate the z-score by using the mean absolute deviation (MeanAD) rather than the median absolute deviation (MAD) makes the method robust. For short series with very little variation, this prevents a small deviation from already being considered an outlier. However, there are also examples where this strategy is less successful. Figure 5 shows a measurement series of ammonium analyses with two clearly anomalous measured values. From the figure, it seems as though both measurements are potential outliers, but the method flags only the first one. This is because the second measurement has a z-score of 3.2 and thus just fails to meet the outlier criterion of a z-score of 3.5.

**Fig. 6:**
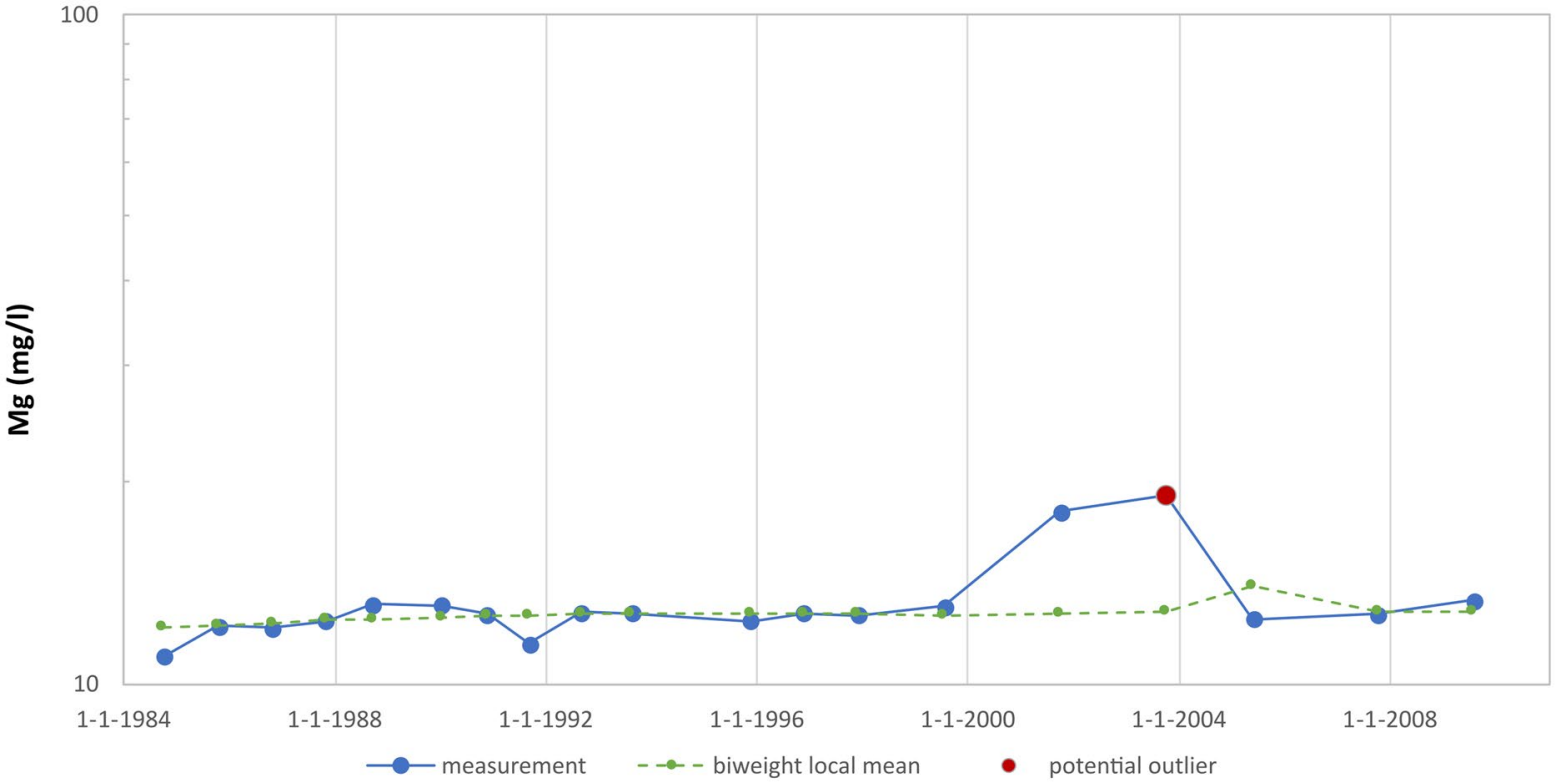
Measurement series for magnesium (well 65) with low variation, as a result of which a small deviation is, rightly or wrongly, still considered an outlier

Another tricky case is when the measurement series exhibits very little variation for most of the time. Figure 6 shows a measurement series for magnesium analyses in which the measured values fluctuate around 12 mg/l, with higher values of 18 mg/l and 19 mg/l only measured in respectively 2001 and 2003. Due to the small variation, the standard deviation is below the threshold value ${\sigma }_{\text{min}}$. The z-score has therefore been determined using the threshold value. Nevertheless, the 2003 measurement appears to have a z-score just above 3.5 and so is identified as a potential outlier. The 2001 measurement is just below the threshold and is not flagged as an outlier. However, it is questionable whether the 2003 measured value is indeed an outlier. This will need to be determined on the basis of additional data and/or additional research.

**Fig. 7:**
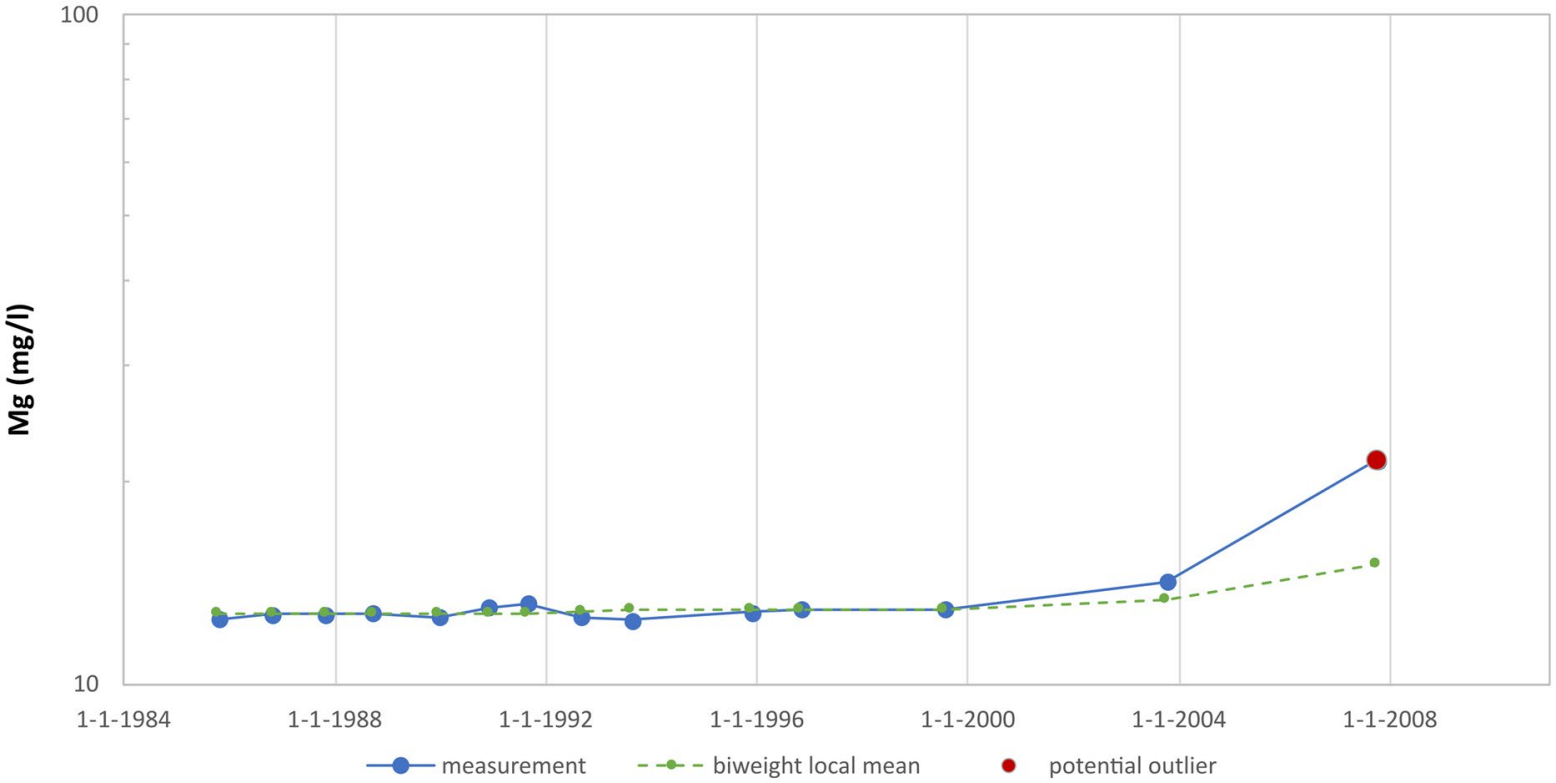
Measurement series for magnesium (well 55), trending upwards at the end of the series

Finally, Fig. 7 shows a measurement progression for magnesium that the method also has difficulty with. It concerns a rising trend at the end of a measurement series (and a flat trend at the beginning of the measurement series). Because the local mean of the last measured value in a series is calculated using only the preceding measurements (and therefore is not central), it is less reliable, which increases the likelihood that the last measurement will be flagged as an outlier even though it might not be one. It is therefore recommended to consider the last measurement in a measurement series as a provisional potential outlier. Whether it really is an outlier can be determined only after several subsequent measurements become available.

### Discussion

The method presented in this paper provides a procedure for automatically checking large datasets of groundwater quality for potential outliers. It gives reliable results, not only for high outliers but also for more subtle outliers near the detection limit. The log-transformation plays a crucial role here, enabling low concentrations with values of tenths and hundredths of mg/l or µg/l to be separated from each other. This gives the method added value over outlier detection methods that do not use log-transformation (Adikaram et al., 2015; Wang et al., 2020).

It is important to note that the method identifies potential outliers and that additional checks are often needed to formally designate them as “outliers” in a database of groundwater quality analyses. Once the potential outliers have been detected, additional checks can be performed for each potential outlier to determine whether it really is an outlier or that the measured value is correct. Additional indications include a significant difference in ion balance (electroneutrality condition) or a significant difference between measured and calculated electrical conductivity, which are general indications of analysis errors. After considering such indicators, it is possible to be more certain which are the actual outliers. However, such checks do not work for trace elements that do not contribute substantially to the ion balance. In such cases, expert opinion is important, possibly supplemented by knowledge of the range in concentrations within the monitoring area. Using the latter, only extreme outliers can be designated, however.

The decision to make the method robust has a downside: the method may miss out potential outliers if the measurement series has multiple sequential outliers. Future research could aim to make further improvements in this regard. It is already possible to adjust the criteria defined in the “The parameter values used” section when applying the method. In this paper, a z-score criterion of 3.5 was used. Choosing a less stringent criterion would result in more measurements qualifying as potential outliers, but then more emphasis would be placed on testing these outliers by e.g. visual inspection or ion balances.

## Conclusions

A flexible method has been devised for detecting outliers in groundwater quality measurement series. It has been developed for datasets with a wide variety of measurement series. The concentrations in a series may vary widely or moderately, may or may not have a temporal trend, and the series may contain measured values below the detection limit. Moreover, the detection limit can vary over time. The method has proved to be sensitive and has detected outliers at the top and bottom of the regular measurement range and around the detection limit. Not unexpectedly, because it is able to analyse such a variety of measurement series, the method does not give results that are 100% satisfactory. Measured values identified by the method as potential outliers will therefore always need to be further assessed based on expert knowledge, consistency with other measurement data and/or additional research. Furthermore, outliers at the end of a measurement series should always be considered “provisional” since an as yet unknown upward or downward trend may follow. Only after new measurements become available can a final judgement be made. Potential outliers at the beginning of a measurement series will need to be assessed by an expert using additional information.

Although developed for the analysis of groundwater quality data, the method presented in this paper can also be applied to other data with similar characteristics (short series, presence of trends and non-detects), in earth and environmental sciences or other sciences.

## Data Availability

The datasets analysed during this study and scripts used for these analyses are available from the corresponding author upon request.
